# The physiological effects of breath‐holding during high‐intensity exercise

**DOI:** 10.14814/phy2.70437

**Published:** 2025-06-25

**Authors:** Jeremy Walsh, James L. Ramsey, Nasimi A. Guluzade, Robin Faricier, Daniel A. Keir, Glen R. Belfry

**Affiliations:** ^1^ School of Kinesiology Western University London Ontario Canada; ^2^ Lawson Health Research Institute London Ontario Canada; ^3^ Toronto General Hospital Research Institute, Toronto General Hospital Toronto Ontario Canada

**Keywords:** apnea, breath‐holding, exercise, high‐intensity

## Abstract

The purpose of this study was to determine the physiological effects of breath‐holding during high‐intensity exercise. Twenty participants (age: 23 ± 4 years, 10 females) performed 20 s sprints of simultaneous arm and leg ergometry exercise, under free‐breathing (FB) and breath‐holding (BH) conditions. Identical power outputs were sustained for both breathing conditions. Heart rate was significantly higher under the breath‐holding condition during exercise (FB = 118 ± 12 bpm versus BH = 131 ± 14 bpm; *p* < 0.001) but lower for 5 min post‐exercise (FB = 110 ± 16 bpm, BH = 102 ± 15 bpm, *p* = 0.003). Systolic blood pressure was higher under the breath‐holding condition post‐exercise (FB = 142 ± 18 mmHg, BH = 151 ± 15 mmHg, *p* = 0.03). Muscle deoxygenated hemoglobin was unchanged between conditions during exercise (FB = 2.7 ± 1.7 μM, BH = 2.6 ± 1.7 μM, *p* = 0.37) and no difference in post‐exercise blood lactate concentration was observed between conditions (FB = 9.3 ± 3.5 mmol.L^−1^, BH = 8.2 ± 2.5 mmol.L^−1^, *p* = 0.15). End‐tidal partial pressure of oxygen was reduced (FB = 111 ± 11 mmHg, BH = 76 ± 14 mmHg, *p* < 0.001) and oxygen uptake was increased (FB = 2.36 ± 0.63 L.min^−1^, BH = 4.52 ± 0.73 L.min^−1^, *p* < 0.001) under the breath‐holding condition immediately post‐exercise. Exercise‐induced tachycardia prevailed over apnea‐induced bradycardia during exercise, but residual effects of breath‐holding were evidenced by the observed bradycardia during recovery. These data suggest that intrinsic oxygen stores were sufficient to sustain the aerobic energy contribution during 20 s high‐intensity exercise while breath‐holding.

## 1 INTRODUCTION

Breath‐holding is performed in different sport and work‐related contexts such as swimming, free diving, artistic swimming, underwater rugby and hockey, sponge and pearl harvesting, and spear fishing (Dujic & Breskovic, 2012), as well as during water rescue operations. The 50 m freestyle sprint is the fastest and shortest swimming race, taking the best swimmers just over 20 s to complete (Pyne & Sharp, 2014). Turning the head to breathe during freestyle swimming has been shown to have an adverse effect on stroke mechanics, as well as increasing drag, potentially compromising performance (McCabe et al., 2015; Zamparo et al., 2020). Thus, during the 50 m sprint event, swimmers often forego breathing to improve performance (McCabe et al., 2015). Although the energy required to provide the propulsive power for 50 m sprint swimming is provided mainly by high‐energy phosphates and the anaerobic glycolytic energy systems (Pyne & Sharp, 2014), the aerobic energy system contributes ~15% of the total energy required for maximal efforts of this duration (Capelli et al., 1998; Péronnet & Thibault, 1989). During breath‐holding, also known as apnea, oxygen (O_2_) cannot be sourced from the atmosphere and aerobic energy production relies on intrinsic O_2_ stores in the lungs from inspiration prior to apnea, in the blood, dissolved and bound to hemoglobin, and in muscle, bound to myoglobin (Panneton, 2013). As these stores are finite and not replenished during apnea, these may become depleted, resulting in reduced O_2_ availability for aerobic energy production as apnea progresses (Nishiyasu et al., 2012).

Physiological adaptations have evolved to conserve O_2_ and redistribute blood flow to the vital organs to mitigate its reduced availability during apnea (Bain et al., 2018; Foster & Sheel, 2005). These O_2_‐conserving responses include bradycardia, reduced cardiac output, peripheral vasoconstriction, increased blood pressure (BP), and splenic contraction and are collectively known as the “diving response” (Foster & Sheel, 2005; Gooden, 1994). During apnea, reduced lung movement leads to reduced stretch on the lungs with subsequent reduction in activity of the slowly adapting pulmonary stretch receptors and so the cardiac vagal motoneurons can exert their influence leading to bradycardia and reduced cardiac output (Bain et al., 2018). Bradycardia reduces blood supply to the periphery and there is also reduced myocardial O_2_ consumption during bradycardia, both of which conserve O_2_ stores (Alboni et al., 2011). While increased parasympathetic activity leads to bradycardia during apnea, increased sympathetic activity leads to peripheral vasoconstriction which reduces O_2_ consumption in the periphery and redistributes blood flow, prioritizing O_2_ rich blood flow to the brain (Alboni et al., 2011; Bain et al., 2018; Foster & Sheel, 2005). This peripheral vasoconstriction leads to an increase in BP which contributes to the bradycardia via the baroreflex (Perini et al., 2008). Furthermore, as the apnea is sustained, hypoxaemia will ensue, leading to the stimulation of peripheral chemoreceptors resulting in accentuated bradycardia and vasoconstriction (Alboni et al., 2011; Foster & Sheel, 2005).

This O_2_‐conserving response to apnea is contradictory to the O_2_‐demanding response to exercise, which includes tachycardia, increased cardiac output, and peripheral vasodilation (Di Giacomo et al., 2021; Nobuhiro et al., 2024; Tocco et al., 2013). Therefore, when breath‐holding occurs during exercise, these conflicting stimuli emerge simultaneously (Bouten et al., 2021), raising the question: what is the net effect of the contradictory responses to these stimuli? If the apnea response prevails, reduced O_2_ availability at the exercising muscles may compromise aerobic energy production and impair performance (Ichinose et al., 2018; Nishiyasu et al., 2012). However, this potential apnea‐induced O_2_ deficit may be mitigated by increased reliance on the intrinsic O_2_ stores for aerobic energy production or increased contributions from anaerobic energy systems to meet the energy demand required to sustain the exercise (Ferretti, 2001; Lindholm & Linnarsson, 2002).

The apnea response has been shown to prevail over the exercise response during low‐intensity exercise, and despite its O_2_‐conserving effects, the exercise can be sustained through increased reliance on intrinsic O_2_ stores and anaerobic glycolysis (Alboni et al., 2011; Andersson et al., 2002, 2004; Bergman et al., 1972; Ichinose et al., 2018; Lindholm et al., 1999, 2002; Lindholm & Linnarsson, 2002; Nishiyasu et al., 2012). Increased reliance on intrinsic O_2_ stores has been indicated by reduced muscle O_2_ saturation (S_m_O_2_) and increased muscle deoxygenated hemoglobin ([HHb]) as measured by near‐infrared spectroscopy (NIRS) (Bouten et al., 2021), and reduced partial pressures of O_2_ (Ferretti et al., 1991) and increased O_2_ uptake (V̇O_2_) post‐apnea (Linér & Linnarsson, 1994). Increased reliance on anaerobic glycolysis has been indicated by increased blood lactate concentration ([La^−^]) (Andersson et al., 2004). However, it is less clear as to which of the exercise or apnea responses prevails during high‐intensity exercise, such as sprint swimming, considering the increased parasympathetic withdrawal and sympathetic drive of high‐intensity exercise, and whether its increased energy demands can be sufficiently met to sustain the exercise (Asmussen & Kristiansson, 1968; Guimard et al., 2014, 2017, 2018). Furthermore, the residual effects of breath‐holding during exercise, post‐apnea, have rarely been reported. While bradycardia during low‐intensity exercise has been well‐established, the time course for the return of heart rate (HR) to pre‐apnea levels has been variable, for example, “immediately” post‐apnea (Bouten et al., 2020), “once breathing resumed” (Bergman et al., 1972), approximately 25 s following the end of apnea (Bouten et al., 2021), by the final 30 s of the first min post‐apnea (Breskovic et al., 2011) and “1‐2 min after the end of intervention” (Espersen et al., 2002). In one study, a bradycardia compared to pre‐exercise baseline HR was observed 2.5‐ and 5‐min post‐apnea and HR had returned to baseline by 15 min post‐apnea (Brown et al., 2024). The residual effects of breath‐holding during high‐intensity exercise have not been reported.

Therefore, the purpose of this study was to determine the effects of breath‐holding during high‐intensity exercise. Without a swimming flume or underwater compatible gas mass spectrometry and muscle NIRS collection systems available, this initial, exploratory, land‐based study used simultaneous arm and leg ergometry exercise to mimic the muscular demands of swimming. Twenty s bouts were compared under free‐breathing and breath‐holding conditions. The objectives were to determine the effects of breath‐holding on HR, BP, muscle oxygenation, [La^−^], and gas exchange and ventilatory variables during and post‐exercise, to establish whether the exercise or apnea response would prevail, whether O_2_ availability at the exercising muscles would be compromised and whether there would be any residual effects of breath‐holding post‐apnea. It was hypothesized that (1) the apnea response would prevail, indicated by bradycardia and increased BP, compared to free‐breathing, (2) O_2_ availability would be compromised by breath‐holding, as reflected by reduced S_m_O_2_ and increased [HHb] during exercise, with increased [La^−^] and reduced end‐tidal partial pressure of O_2_ (P_et_O_2_) and increased V̇O_2_ post‐exercise, compared to free‐breathing, and (3) effects of breath‐holding would resolve quickly once breathing resumed.

## 2 MATERIALS AND METHODS

### 2.1 Participants

Twenty adults (mean ± standard deviation (SD) age: 23 ± 4 years, height: 174 ± 10 cm, and body mass: 69 ± 13 kg, 10 females) volunteered to participate in this study (Table 1). All procedures were approved by the Western University Research Ethics Board for Health Sciences Research Involving Human Participants (REB #118179) and were in accordance with the 1964 Helsinki declaration and its later amendments or comparable ethical standards. All participants provided written informed consent and were healthy, recreationally active (active 1–3 times per week), and nonsmokers. None of the participants were taking any medications that would affect the cardiorespiratory or hemodynamic responses to exercise.

**TABLE 1:** Participant characteristics and power outputs used during exercise.

Sex	Participant	Age	Height	Mass	Power output (W)		
		(years)	(cm)	(kg)	Arms	Legs	Total
Males	1	24	180	78	459	184	642
	2	22	186	82	482	193	675
	3	23	190	100	588	235	824
	4	25	185	87	512	205	716
	5	21	195	86	506	202	708
	6	24	180	63	371	148	519
	7	20	178	73	429	172	601
	8	19	175	79	465	186	651
	9	19	170	67	394	158	552
	10	33	174	62	365	146	511
	Mean	23	181	78	457	183	640
	SD	4	8	12	70	28	98
Females	1	20	165	61	359	144	502
	2	24	165	67	394	158	552
	3	21	152	46	271	108	379
	4	19	170	61	359	144	502
	5	19	168	68	400	160	560
	6	19	168	63	371	148	519
	7	20	168	53	312	125	436
	8	29	170	65	382	153	535
	9	22	168	63	371	148	519
	10	31	173	63	371	148	519
	Mean	22	167	61	359	144	502
	SD	4	6	7	39	16	55

Abbreviations: cm, centimeters; kg, kilograms; SD, standard deviation; W, watts.

### 2.2 Protocol

An electromagnetically‐braked cycle ergometer (Velotron; RacerMate, Seattle, WA) was used for leg ergometry and an adapted cycle ergometer (Lode Corival 400; Groningen, Netherlands) was used for arm ergometry. At least 48 h prior to testing, subjects attended a familiarization session in which they simultaneously pedaled and arm cranked under free‐breathing and breath‐holding conditions. The ergometer power output (PO) for the legs was calculated as 0.075 kg per kg body mass (BM) at a cadence of 80 revolutions per minute (rpm) (Zupan et al., 2009) and 0.04 kg per kg BM at a cadence of 60 rpm for the arms (Forbes et al., 2014). The ergometers were set to pedaling speed independent mode, so regardless of cadence the PO was unchanged. Mean ± SD PO for the legs and arms were 408 ± 75 Watts (W) and 163 ± 30 W, respectively, for a total PO of 571 ± 105 W (Table 1).

Participants were tested on two occasions, with a minimum of 48 h between sessions. One test was performed while free‐breathing; the other was performed while breath‐holding. The order of testing was randomized. Prior to each test, participants were advised whether they would be exercising under free‐breathing or breath‐holding conditions. For breath‐holding, they were instructed to take a deep inspiration during the 3 s countdown to the start of exercise and to endeavor not to exhale again until instructed to do so by the investigator at the end of the exercise bout. Participants were seated on the cycle ergometer with their legs resting on stools on either side of the pedals and their hands resting on their knees for 3 min prior to the exercise bout. Thirty second prior to the start of exercise, the investigator manually turned the cycle ergometer pedals for 10 s to get the flywheel moving at ~80 rpm. Twenty second prior to the onset of exercise, the participant's feet were placed on the pedals. Fifteen s prior to the start of exercise the investigator manually turned the arm ergometer handgrips for 10 s to get the flywheel moving at ~60 rpm. Five second prior to the onset of exercise, the participant's hands were placed on the handgrips. Three second prior to the onset of exercise, participants were given a second‐by‐second countdown and, at the onset of exercise, participants pedaled the legs and cranked the arms for 20 s. At the end of the exercise bout, participants were instructed to remain seated on the cycle ergometer, with their legs resting on the stools on either side of the pedals and their hands resting on their knees for 15 min.

### 2.3 Measurements

#### 2.3.1 Heart rate and blood pressure

Heart rate was measured by a chest strap monitor (Polar H10, Polar Electro Oy, Kempele, Finland) and recorded on a second‐by‐second basis by a Polar Beat application (version 3.5.6., Polar Electro Oy, Kempele, Finland). Systolic and diastolic BP were measured using an automatic sphygmomanometer (Patient Monitor, PM80D, Guandong, China) 4 min pre‐ and immediately post‐exercise.

#### 2.3.2 Muscle oxygenation

Muscle oxygenation was monitored continuously using NIRS (Oxiplex TS, model 95,205, ISS, Champaign, IL). NIRS probes were placed on the left vastus lateralis (midway between the lateral epicondyle and greater trochanter of the femur) and triceps brachii (midway between the acromion of the scapula and the olecranon of the ulna) muscles. An elastic strap secured each probe in place. An optically dense, black vinyl sheet was placed over each probe to prevent the exposure to extraneous light. The thigh and arm probes were wrapped with elastic bandages to further minimize the intrusion of extraneous light and movement. NIRS measurements were collected second‐by‐second from 3 min pre‐ to 15 min post‐exercise. [HHb] and oxygenated hemoglobin concentration ([HbO_2_]) were measured, whereas total hemoglobin concentration ([THb]) and S_m_O_2_ were derived with this apparatus. [THb] was calculated as the sum of [HHb] and [HbO_2_], and S_m_O_2_ as the percentage of [HbO_2_] to [THb].

#### 2.3.3 Lactate

Blood [La^−^] from the left index finger was measured 3 min pre‐exercise and 3 min post‐exercise, and the samples were immediately analyzed by SensLab GmbH Lactate SCOUT (Leipzig, Germany) capillary lactate analyzer.

#### 2.3.4 Gas exchange and ventilatory variables

Gas exchange and ventilatory variables (V̇O_2_, carbon dioxide (CO_2_) production (V̇CO_2_), ventilation (V̇_E_), P_et_O_2_, and end‐tidal partial pressure of CO_2_ (P_et_CO_2_)) were measured breath‐by‐breath by a metabolic cart (Quark, CPET; Cosmed, Rome, Italy). Briefly, inspired and expired volume rates were measured by a low‐dead‐space turbine after being calibrated with a syringe of known volume (3 L). Fractional concentrations of inspired and expired O_2_ and CO_2_ for each breath were assessed by gas analyzers that were calibrated before each test using a gas mixture of known concentration.

### 2.4 Data analyses

Before analyses, all NIRS and gas exchange and ventilatory data were processed by removing aberrant data points identified as those positioned more than three SD from the local mean using OriginPro, version 2023b (OriginLab Corporation, Northampton, MA, USA). NIRS data were adjusted to baseline values, using the 60 s average from 90 s to 30 s before the onset of exercise as the baseline due to the movement of the legs and arms onto the pedals and handgrips in the final 30 s before exercise that impacted the baseline data. Changes in S_m_O_2_, [HHb] and [THb] respective to their baseline values (ΔS_m_O_2_, Δ[HHb] and Δ[THb]) were calculated for the vastus lateralis and triceps brachii muscles. As well as analyzing the individual muscles, data from both muscles were averaged and analyzed as combined muscular ΔS_m_O_2_, Δ[HHb] and Δ[THb]. Breath‐by‐breath gas exchange and ventilatory data were linearly interpolated on a second‐by‐second basis. All second‐by‐second data were bin averaged into 5 s, 20 s, and 1 min bins for graphing and statistical purposes.

### 2.5 Statistical analyses

Data are presented as mean ± SD. Paired *t*‐tests were used to determine whether there were any differences in the means of the dependent variables between free‐breathing and breath‐holding conditions at baseline and during exercise. For paired *t*‐tests, Cohen's *d* was used for effect sizes, interpreted as small (*d* > 0.2), moderate (*d* > 0.5), or large (*d* > 0.8). Three‐way repeated measures analysis of variance (ANOVA) was used to determine whether there were any interactions between the main effects of sex, condition (free‐breathing and breath‐holding), and time. For the time factor, the baseline measure and four 5 s bins of the 20 s exercise period were used for HR and muscle oxygenation variables, and pre‐ and post‐ exercise measures were used for BP and [La^−^]. As gas exchange and ventilatory data were not available during breath‐holding, the 5 s bins immediately pre‐ and post‐exercise were used for V̇O_2_, V̇CO_2_, and V̇_E_, and the final breath pre‐exercise and the first breath post‐exercise were used for P_et_O_2_ and P_et_CO_2_. To determine the latent effects of breath‐holding during exercise, three‐way repeated measures ANOVA was used to determine whether there were any interactions between the main effects of sex, condition and time, post‐exercise, with 1 min bins for the first 5 min of the recovery period used as the time factor for all variables.

Data were assessed for normality using the Shapiro–Wilk test. Mauchly's test of sphericity was used to determine whether the variances among the differences of all levels of the independent variables were equal. When the assumption of sphericity was violated, the Greenhouse–Geisser correction was used. Effect sizes for ANOVA are presented as partial eta squared ($\eta_{p}^{2}$) and interpreted as small ($\eta_{p}^{2}$ = 0.01), medium ($\eta_{p}^{2}$ = 0.06), or large ($\eta_{p}^{2}$ = 0.14). Pairwise comparisons with Bonferroni corrections were used when significant interaction or main effects were found. All statistical analyses were performed using IBM SPSS Statistics version 29.0.2.0 (IBM Corp., Armonk, N.Y., USA). Statistical significance was accepted at a level of *p* < 0.05.

## 3 RESULTS

There were no significant differences between free‐breathing and breath‐holding conditions for any of the variables at baseline (Table 2). Although V̇O_2_, V̇CO_2_, V̇_E_, and Δ[THb] were greater in males than females, there were no interactions between the effects of sex and condition and time, or sex and condition, for any variables, that is, there were no differences in the effects of breath‐holding between males and females (Table 3). Muscle oxygenation findings were not different between vastus lateralis and triceps brachii muscles, and so the combined muscle oxygenation data are presented here.

**TABLE 2:** Baseline measures of heart rate and gas exchange and ventilatory variables.

	Baseline		
	FB	BH	*p*‐value
Heart rate (bpm)	82 ± 11	85 ± 14	0.10
V̇O_2_ (L.min^−1^)	0.40 ± 0.07	0.41 ± 0.07	0.25
V̇CO_2_ (L.min^−1^)	0.35 ± 0.06	0.37 ± 0.06	0.31
V̇_E_ (L.min^−1^)	12.9 ± 2.2	13.7 ± 2.5	0.09
P_et_O_2_ (mmHg)	110 ± 3	111 ± 4	0.15
P_et_CO_2_ (mmHg)	32 ± 2	32 ± 3	0.35

*Note*: Data presented are mean ± standard deviation of the 180 s baseline period for heart rate and gas exchange and ventilatory variables. *n* = 20.

Abbreviations: BH, breath‐holding; bpm, beats per minute; FB, free‐breathing; L.min^−1^, liters per minute; mmHg, millimeters of mercury; P_et_CO_2_, end‐tidal partial pressure of carbon‐dioxide; P_et_O_2_, end‐tidal partial pressure of oxygen; V̇CO_2_, rate of carbon dioxide production; V̇_E_, minute ventilation; V̇O_2_, rate of oxygen uptake.

**TABLE 3:** Main effects of sex, and interactions between the effects of sex, conditions and time.

	Main effect of sex			Sex × condition × time interaction
	Males	Females	*p*	*p*
Exercise				
Heart rate (bpm)	113 ± 12	119 ± 9	0.24	0.56
∆S_m_O_2_ (%)	−3.8 ± 1.7	−5.3 ± 1.9	0.08	0.84
∆[THb] (μM)	−0.4 ± 1.6	−0.8 ± 0.9	0.11	0.61
∆[HHb] (μM)	2.3 ± 1.2	2.0 ± 1.5	0.61	0.76
V̇O_2_ (L.min^−1^)	2.26 ± 0.3	1.92 ± 0.3	0.007^a^	0.46
V̇CO_2_ (L.min^−1^)	1.90 ± 0.3	1.50 ± 0.2	0.001^a^	0.65
V̇_E_ (L.min^−1^)	59 ± 12	47 ± 8	0.01^a^	0.93
P_et_O_2_ (mmHg)	102 ± 8	102 ± 3	0.14	0.74
P_et_CO_2_ (mmHg)	39 ± 4	36 ± 3	0.07	0.55
Systolic BP (mmHg)	137 ± 12	134 ± 8	0.57	0.96
Diastolic BP (mmHg)	77 ± 8	77 ± 4	0.76	0.88
Lactate (mmol.L^−1^)	5.2 ± 1.5	5.5 ± 1.1	0.97	0.09
Recovery				
HR (bpm)	108 ± 19	104 ± 8	0.58	0.43
∆S_m_O_2_ (%)	2.0 ± 2.6	1.8 ± 1.8	0.82	0.42
∆[THb] (μM)	5.8 ± 2.1	2.3 ± 1.6	<0.001^a^	0.70
∆[HHb] (μM)	0.2 ± 1.1	−0.1 ± 0.4	0.36	0.34
V̇O_2_ (L.min^−1^)	1.07 ± 0.2	0.80 ± 0.1	0.003^a^	0.46
V̇CO_2_ (L.min^−1^)	1.6 ± 0.3	1.1 ± 0.2	0.002^a^	0.72
V̇_E_ (L.min^−1^)	47 ± 10	35 ± 6	0.005^a^	0.38

*Note*: Data presented are mean ± standard deviation. *n* = 10 males, *n* = 10 females.

Abbreviations: [HHb], deoxygenated hemoglobin; [THb], total hemoglobin; BP, blood pressure; bpm, beats per minute; L.min^−1^, liters per minute; mmHg, millimeters of mercury; mmol.L^−1^, millimols per liter; P_et_CO_2_, end‐tidal partial pressure of carbon‐dioxide; P_et_O_2_, end‐tidal partial pressure of oxygen; S_m_O_2_, muscle oxygen saturation; V̇CO_2_, minute rate of carbon dioxide production; V̇_E_, minute ventilation; V̇O_2_, minute rate of oxygen uptake; Δ, change from baseline; μM, micromoles.

^a^ Significant difference between males and females.

### 3.1 Exercise

#### 3.1.1 Heart rate

**FIGURE 1:**
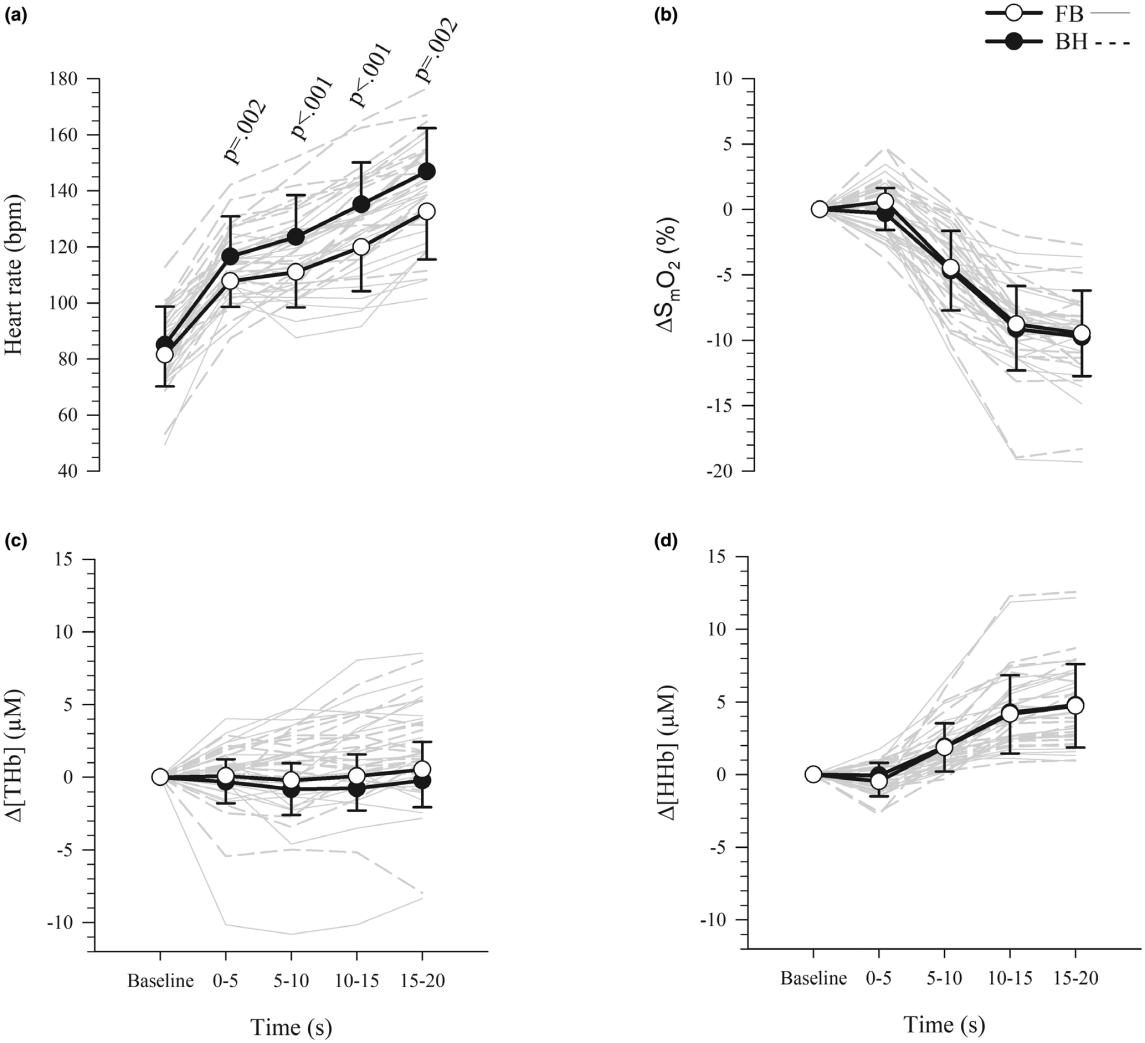
Heart rate and muscle oxygenation during exercise. Data presented are mean (symbols) ± standard deviation (error bars) for the baseline period and in 5 s bins for the 20 s exercise period for (a) heart rate, and changes from baseline in (b) muscle oxygen saturation (ΔS_m_O_2_), (c) total hemoglobin (Δ[THb]), and (d) deoxygenated hemoglobin (Δ[HHb]). Gray lines represent individual responses. *n* = 20 for all panels. μM, micromoles; BH, breath‐holding (black circles); bpm, beats per minute; FB, free‐breathing (white circles).

Heart rate was significantly higher during exercise under the breath‐holding condition (free‐breathing [FB] = 118 ± 12 bpm, breath‐holding [BH] = 131 ± 14 bpm; *p* < 0.001, *d* = 0.98), and there was a significant interaction between the effects of condition and time (*F*_(1.9,34.3)_ = 4.9, *p* = 0.02, $\eta_{p}^{2}$ = 0.21), that is, HR was not significantly different between conditions at baseline (*p* = 0.10) but was significantly higher under the breath‐holding condition for each 5 s bin of the 20 s exercise period (*p* ≤ 0.002) (Figure 1a).

#### 3.1.2 Blood pressure

**FIGURE 2:**
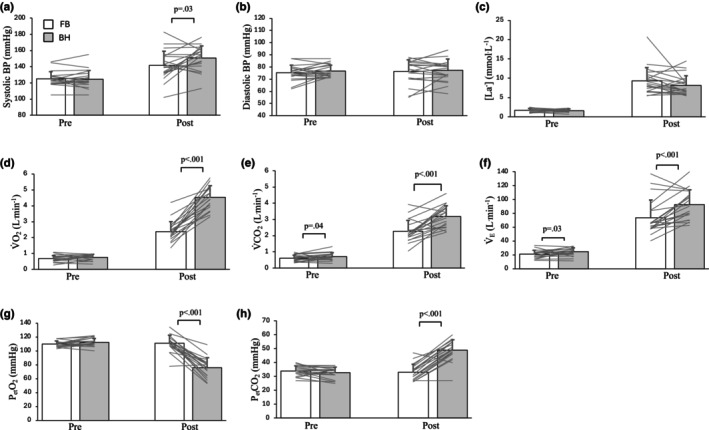
Blood pressure, lactate, and gas exchange and ventilatory data. Data presented are mean (bars) ± standard deviation (error bars) of pre‐ and post‐exercise measures for (a) systolic and (b) diastolic blood pressures (BP), and (c) blood lactate; the 5 s immediately pre‐ and post‐exercise for (d) rate of oxygen uptake (V̇O_2_), (e) rate of carbon dioxide production (V̇CO_2_), and (f) minute ventilation (V̇_E_); and the final breath pre‐ and first breath post‐exercise for end‐tidal partial pressures of (g) oxygen (P_et_O_2_) and (h) carbon‐dioxide (P_et_CO_2_). Lines represent individual responses. *n* = 20 for all panels. BH, breath‐holding (gray bars); FB, free‐breathing (white bars); L.min^−‐1^, liters per minute; mmHg, millimeters of mercury; mmol.L^−1^, millimols per liter; Post, post‐exercise; Pre, pre‐exercise.

There was a significant interaction between the effects of condition and time on systolic BP (*F*_(1,18)_ = 6.2, *p* = 0.02, $\eta_{p}^{2}$ = 0.26; Figure 2a), which was not significantly different between conditions pre‐exercise (FB = 125 ± 9 mmHg, BH = 125 ± 11 mmHg; *p* = 0.91) but was significantly higher under the breath‐holding condition post‐exercise (FB = 142 ± 18 mmHg, BH = 151 ± 15 mmHg; *p* = 0.03). There was no significant interaction between the effects of condition and time on diastolic BP (*F*_(1,18)_ = 0.02, *p* = 0.88, $\eta_{p}^{2}$ = 0.001; Figure 2b), that is, there were no significant differences in diastolic BP between conditions pre‐ or post‐exercise and there were no main effects of condition or time.

#### 3.1.3 Muscle oxygenation

There were no significant differences in ΔS_m_O_2_ (FB = −6 ± 2%, BH = −6 ± 3%; *p* = 0.29, *d* = 0.17), Δ[THb] (FB = −0.5 ± 1.9 μM, BH = 0.1 ± 2.2 μM; *p* = 0.15, *d* = 0.31), or Δ[HHb] (FB = 2.7 ± 1.7 μM, BH = 2.6 ± 1.7 μM; *p* = 0.37, *d* = 0.09) between conditions during exercise. There were no interactions between the effects of condition and time on ΔS_m_O_2_ (*F*_(2.3,41.3)_ = 0.8, *p* = 0.49, $\eta_{p}^{2}$ = 0.04; Figure 1b), Δ[THb] (*F*_(1.8,32.5)_ = 1.2, *p* = 0.31, $\eta_{p}^{2}$ = 0.06; Figure 1c), or Δ[HHb] (*F*_(2.4,42.5)_ = 0.9, *p* = 0.45, $\eta_{p}^{2}$ = 0.05; Figure 1d), that is, there were no significant differences between conditions for any of the 5 s bins during exercise, nor were there were main effects of condition. There were main effects of time on ΔS_m_O_2_ (*F*_(1.9,34.1)_ = 126.1, *p* < 0.001, $\eta_{p}^{2}$ = 0.88) and Δ[HHb] (*F*_(1.3,22.5)_ = 49.4, *p* < 0.001, $\eta_{p}^{2}$ = 0.73), which were lower and higher than baseline, respectively, from 5 to 20 s of exercise.

#### 3.1.4 Lactate

There was no significant interaction between the effects of condition and time on [La^−^] (*F*_(1,18)_ = 2.4, *p* = 0.14, $\eta_{p}^{2}$ = 0.12; Figure 2c), that is, there were no significant differences in [La^−^] between conditions pre‐exercise (FB = 1.7 ± 0.5 mmol.L^−1^, BH = 1.6 ± 0.4 mmol.L^−1^; *p* = 0.86) or post‐exercise (FB = 9.3 ± 3.5 mmol.L^−1^, BH = 8.2 ± 2.5 mmol.L^−1^; *p* = 0.15). There was no main effect of condition, but there was a main effect of time, with [La^−^] being higher post‐exercise compared to pre‐exercise (*F*_(1,18)_ = 143.8, *p* < 0.001, $\eta_{p}^{2}$ = 0.89).

#### 3.1.5 Gas exchange and ventilatory variables

There was a significant interaction between the effects of condition and time on V̇O_2_ (*F*_(1,18)_ = 120.3, *p* < 0.001, $\eta_{p}^{2}$ = 0.87), which was not significantly different between conditions pre‐exercise (FB = 0.67 ± 0.2 L.min^−1^, BH = 0.76 ± 0.18 L.min^−1^; *p* = 0.09) but was significantly higher under the breath‐holding condition post‐exercise (FB = 2.36 ± 0.63 L.min^−1^, BH = 4.52 ± 0.73 L.min^−1^; *p* < 0.001) (Figure 2d).

There were significant interactions between the effects of condition and time on V̇CO_2_ (*F*_(1,18)_ = 19.4, *p* < 0.001, $\eta_{p}^{2}$ = 0.52; Figure 2e) and V̇_E_ (*F*_(1,18)_ = 9.1, *p* = 0.008, $\eta_{p}^{2}$ = 0.34; Figure 2f) which were both significantly higher under the breath‐holding condition immediately pre‐exercise (V̇CO_2_: FB = 0.61 ± 0.19 L.min^−1^, BH = 0.72 ± 0.23 L.min^−1^; *p* = 0.04; V̇_E_: FB = 21 ± 6 L.min^−1^, BH = 24 ± 6 L.min^−1^; *p* = 0.03) and post‐exercise (V̇CO_2_: FB = 2.26 ± 0.68 L.min^−1^, BH = 3.17 ± 0.68 L.min^−1^; *p* < 0.001; V̇_E_: FB = 74 ± 25 L.min^−1^, BH = 93 ± 21 L.min^−1^; *p* < 0.001).

There were significant interactions between the effects of condition and time on P_et_O_2_ (*F*_(1,18)_ = 114.2, *p* < 0.001, $\eta_{p}^{2}$ = 0.86; Figure 2g) and P_et_CO_2_ (*F*_(1,18)_ = 111.1, *p* < 0.001, $\eta_{p}^{2}$ = 0.86; Figure 2h), which did not significantly differ between conditions pre‐exercise (P_et_O_2_: FB = 110 ± 4 mmHg, BH = 112 ± 6 mmHg; *p* = 0.06; P_et_CO_2_: FB = 34 ± 4 mmHg, BH = 33 ± 4 mmHg; *p* = 0.09) but P_et_O_2_ was significantly lower (FB = 111 ± 11 mmHg, BH = 76 ± 14 mmHg; *p* < 0.001) and P_et_CO_2_ was significantly higher (FB = 33 ± 6 mmHg, BH = 49 ± 7 mmHg; *p* < 0.001) under the breath‐holding condition, post‐exercise.

### 3.2 Recovery

#### 3.2.1 Heart rate and muscle oxygenation

**FIGURE 3:**
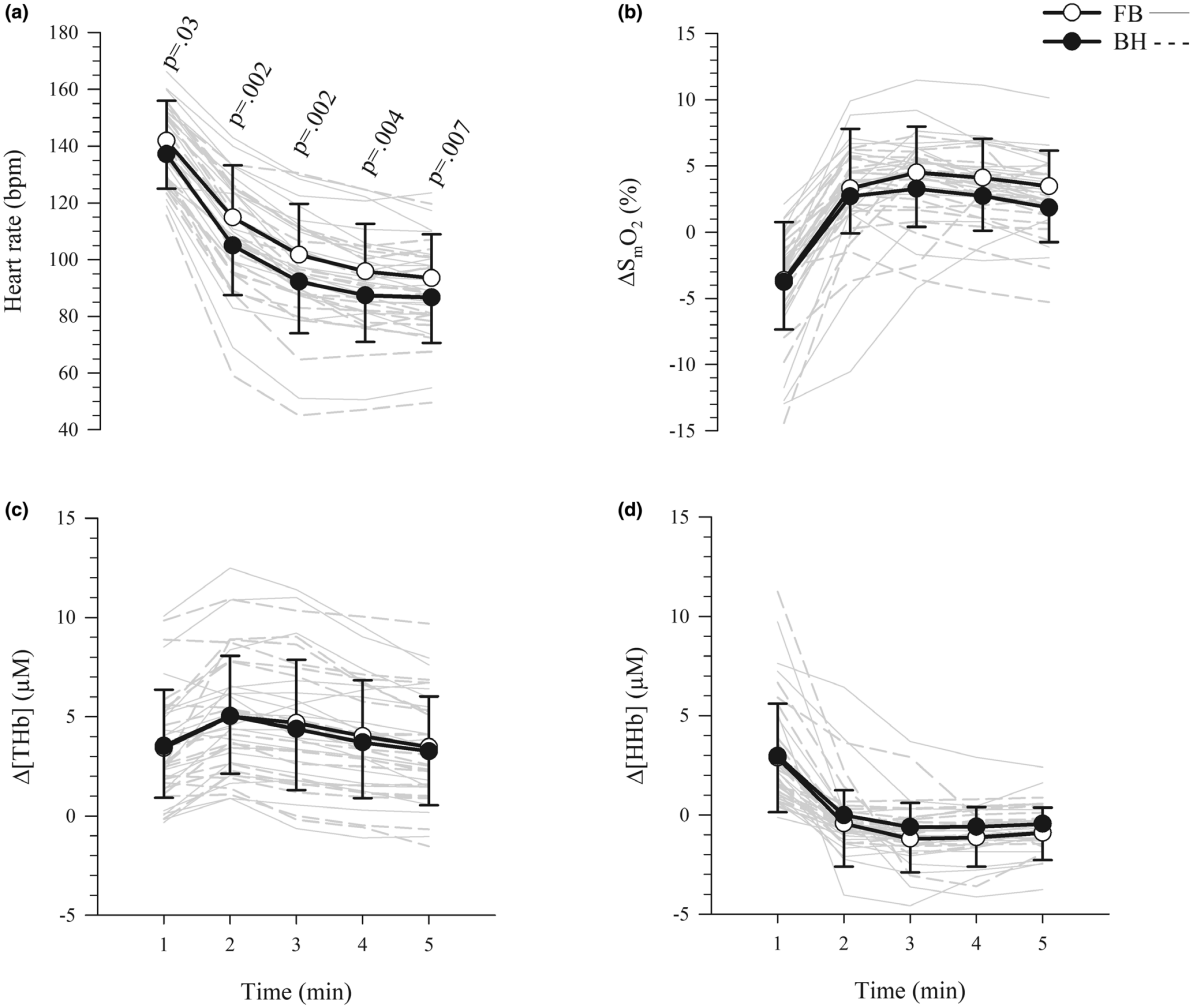
Heart rate and muscle oxygenation during recovery. Data presented are mean (symbols) ± standard deviation (error bars) of the 1 min bins of the first 5 min post exercise for (a) heart rate, and changes from baseline in (b) muscle oxygen saturation (ΔS_m_O_2_), (c) total hemoglobin (Δ[THb]), and (d) deoxygenated hemoglobin (Δ[HHb]). Gray lines represent individual responses. *n* = 20 for all panels. μM = micromoles; BH, breath‐holding (black circles); bpm, beats per minute; FB, free‐breathing (white circles).

During recovery, there was a significant interaction between the effects of condition and time on HR (*F*_(2.1,37.4)_ = 4.0, *p* = 0.03, $\eta_{p}^{2}$ = 0.18; Figure 3a), which was significantly lower under the breath‐holding condition for each min and for the entire 5 min (FB = 110 ± 16 bpm, BH = 102 ± 15 bpm, *p* = 0.003, $\eta_{p}^{2}$ = 0.39). There were no significant interactions between the effects of condition and time on ΔS_m_O_2_ (*F*_(2.1,38.0)_ = 2.1, *p* = 0.14, $\eta_{p}^{2}$ = 0.10; Figure 3b), Δ[THb] (*F*_(1.9,33.4)_ = 0.8, *p* = 0.44, $\eta_{p}^{2}$ = 0.04; Figure 3c) or Δ[HHb] (*F*_(1.9,34.5)_ = 0.9, *p* = 0.41, $\eta_{p}^{2}$ = 0.05; Figure 3d), that is, there were no significant differences between conditions at any min during recovery, nor were there main effects of condition. There were main effects of time on ΔS_m_O_2_ which was lower for the first min compared to the subsequent 4 min (*F*_(1.3,23.7)_ = 46.9, *p* < 0.001, $\eta_{p}^{2}$ = 0.72), Δ[THb] which increased between the first and second min before decreasing continuously from the second through fifth min (*F*_(1.5,27.5)_ = 19.6, *p* < 0.001, $\eta_{p}^{2}$ = 0.52), and Δ[HHb] which was higher for the first min compared to the subsequent 4 min (*F*_(1.2,21.6)_ = 26.5, *p* < 0.001, $\eta_{p}^{2}$ = 0.60).

#### 3.2.2 Gas exchange and ventilatory variables

**FIGURE 4:**
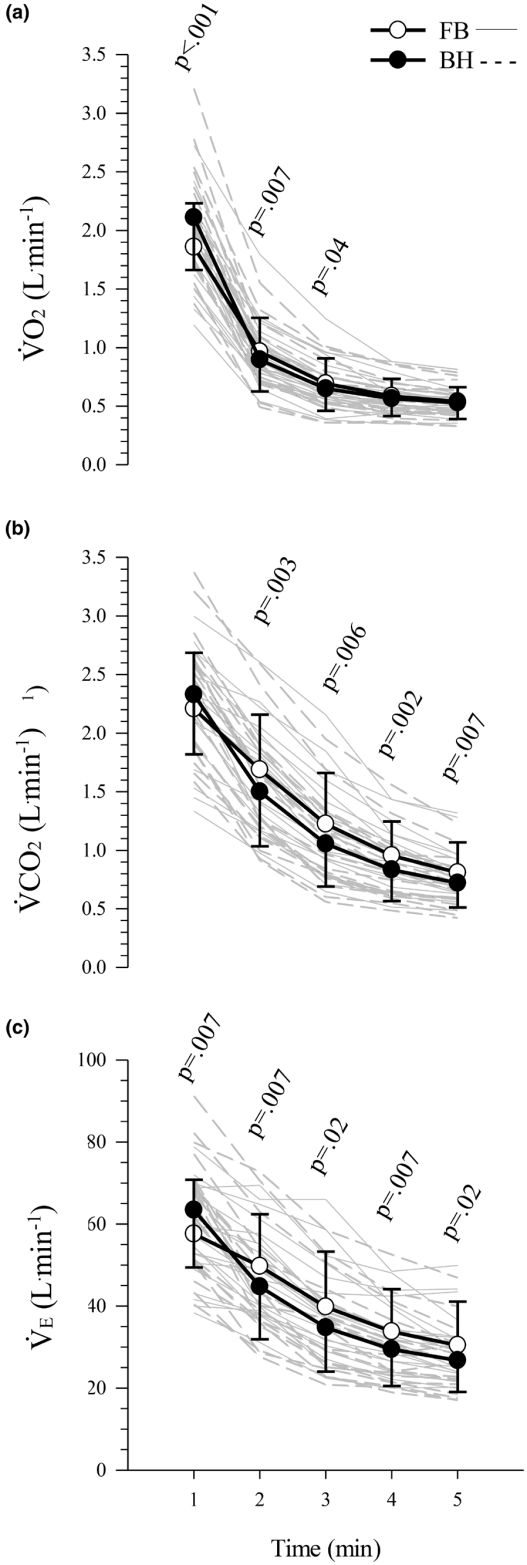
Gas exchange and ventilatory data during recovery. Data presented are mean (symbols) ± standard deviation (error bars) of the 1 min bins of the first 5 min post exercise for (a) rate of oxygen uptake (V̇O_2_), (b) rate of carbon dioxide production (V̇CO_2_), and (c) minute ventilation (V̇_E_). Gray lines represent individual responses. *n* = 20 for all panels. BH, breath‐holding (black circles); FB, free‐breathing (white circles); L.min^−1^, liters per minute.

There was a significant interaction between the effects of condition and time on V̇O_2_ (*F*_(1.6,29.2)_ = 33.2, *p* < 0.001, $\eta_{p}^{2}$ = 0.65; Figure 4a), which was significantly higher under the breath‐holding condition for the first min (FB = 1.86 ± 0.37 L.min^−1^, BH = 2.1 ± 0.44 L.min^−1^; *p* < 0.001) and lower under the breath‐holding condition for the second (FB = 0.97 ± 0.28 L.min^−1^, BH = 0.90 ± 0.26 L.min^−1^; *p* = 0.007) and third min of recovery (FB = 0.69 ± 0.21 L.min^−1^, BH = 0.65 ± 0.18 L.min^−1^; *p* = 0.04).

There was a significant interaction between the effects of condition and time on V̇CO_2_ (*F*_(1.8,33.2)_ = 13.0, *p* < 0.001, $\eta_{p}^{2}$ = 0.42; Figure 4b), which was significantly lower under the breath‐holding condition from the second through fifth min of recovery (FB = 1.17 ± 0.36 L.min^−1^, BH = 1.03 ± 0.32 L.min^−1^; *p* = 0.002).

There was a significant interaction between the effects of condition and time on V̇_E_ (*F*_(2.1,37.5)_ = 22.4, *p* < 0.001, $\eta_{p}^{2}$ = 0.55; Figure 4c) which was significantly higher under the breath‐holding condition for the first min (FB = 58 ± 13 L.min^−1^, BH = 63 ± 14 L.min^−1^; *p* = 0.007) and lower under the breath‐holding condition from the second through fifth min of recovery (FB = 38 ± 11 L.min^−1^, BH = 34 ± 10 L.min^−1^; *p* = 0.006).

## 4 DISCUSSION

The main findings of this study of breath‐holding during high‐intensity exercise were that (1) the exercise response prevailed over the apnea response, as tachycardia was observed during breath‐holding exercise, compared to free‐breathing, (2) O_2_ availability was not compromised by breath‐holding as there was not an increased reliance on O_2_ extraction, or anaerobic glycolysis, as evidenced by the unchanged Δ[HHb] and [La^−^], between breathing conditions, and (3) there were residual effects of breath‐holding during exercise, as bradycardia and increased BP were observed post‐apnea, during recovery, compared to free‐breathing.

### 4.1 Physiological response during exercise

#### 4.1.1 Exercise response versus apnea response

Heart rate was higher during exercise under the breath‐holding condition, compared to free‐breathing (Figure 1a). This contrasts with the well‐established apnea‐induced bradycardia at rest (Bourdas & Geladas, 2024; Bouten et al., 2020; Kiviniemi et al., 2012; Lemaître et al., 2008; Perini et al., 2008; Tocco et al., 2013) and during low‐intensity exercise (Andersson et al., 2002, 2004; Bouten et al., 2021; Breskovic et al., 2011; Guimard et al., 2021; Ichinose et al., 2018; Kjeld et al., 2009; Lindholm et al., 1999, 2002; Lindholm & Linnarsson, 2002; Nishiyasu et al., 2012). The response to apnea during low‐intensity exercise is that of an initial tachycardia (Caspers et al., 2011) due to the sympathetic anticipatory response to apnea and exercise, combined with the effects of the inspiration prior to the breath‐holding (Bouten et al., 2021; Wein et al., 2007). The inspiration prior to the breath‐hold both stimulates lung stretch receptors (which inhibit cardiac vagal nerves) and increases cardiac transmural pressures (which leads to decreased cardiac filling and reduces BP), which increase HR (Andersson & Schagatay, 1998; Cheyne et al., 2018). When breath‐holding is performed during low‐intensity exercise, HR increases for the first 5–10 s due to these stimuli, but bradycardia subsequently prevails due to the reduced activity of pulmonary stretch receptors leading to increased parasympathetic drive (Alboni et al., 2011; Bouten et al., 2021; Lindholm et al., 2002). However, higher exercise intensities and therefore higher sympathetic activity delay the onset of apnea‐induced bradycardia (Wein et al., 2007). In our study of high‐intensity exercise, tachycardia was observed for the entire 20 s breath‐holding period (Figure 1a). It is possible that the increased sympathetic drive of high‐intensity exercise, superimposed on the anticipatory sympathetic effects and parasympathetic withdrawal due to pre‐apnea inspiration, resulted in the initial tachycardia of apnea being sustained for the entire exercise duration. Moreover, this tachycardia, rather than bradycardia, during breath‐holding suggests that the increased sympathetic drive of the high‐intensity exercise predominated over any apnea‐induced sympathetic withdrawal and increased parasympathetic drive. The effects of high‐intensity exercise offset the effects of the apnea response (Tocco et al., 2013). Indeed, there may be an intensity threshold above which exercise tachycardia prevails over apnea‐induced bradycardia. When apnea was performed during steady state cycling, bradycardia occurred when cycling at 20% and 30% of peak power output, but not at 40% and 50% of peak power output suggesting that apnea‐induced bradycardia prevails at lower but not higher intensities (Guimard et al., 2021). This is in keeping with the present study where bradycardia was not evident during 20 s high‐intensity (571 ± 105 W) exercise while breath‐holding.

Notably, breath‐holding time is shorter for higher compared to lower exercise intensities, and the bradycardic response may be limited by an inability to sustain the breath‐hold for long enough for its effects to be observed (Bergman et al., 1972; Guimard et al., 2021; Wein et al., 2007). With increasing cycling intensity, maximal breath‐holding time decreased from 85 s at 40 W to 69 s at 80 W and 62 s at 120 W (Wein et al., 2007). Furthermore, at 40, 80, and 120 W, the onset of bradycardia was after 13, 27, and 27 s, respectively, again suggesting that as duration decreases concomitant to the increased intensity, the bradycardic effect is delayed (Wein et al., 2007). Moreover, it has been suggested that a minimum of 30–40 s in apnea is required to obtain the full development of apnea‐induced bradycardia (Jung & Stolle, 1981; Schagatay et al., 1999). In the present study, our data suggest that the 20 s duration was too short and the intensity too high for apnea‐induced bradycardia to evolve, and that the exercise response dominated over the apnea response during exercise.

#### 4.1.2 Energy production

The identical power outputs sustained under both breathing conditions during the 20 s exercise show that energy availability was not compromised by the apnea. Breath‐holding during 20 s high‐intensity exercise did not result in increased Δ[HHb] or decreased ΔS_m_O_2_ compared to free‐breathing (Figure 1b,d). This demonstrates that O_2_ extraction was unchanged between free‐breathing and breath‐holding conditions in the muscles observed. In contrast, it has been shown that during maximal duration apneas at rest and apneas while exercising at low intensity (30 s breath‐holds while cycling at 25% peak power output), peripheral vasoconstriction occurred within 10 s of the onset of breath‐holding, resulting in reduced blood flow and O_2_ delivery (Bouten et al., 2020, 2021). Continued extraction of O_2_ from this reduced blood flow precipitated decreased S_m_O_2_ and increased [HHb] compared to free‐breathing (Bouten et al., 2020). That this did not occur in our study may be due to the observed tachycardia during breath‐holding (Figure 1a) and increased systolic BP (Figure 2a) which maintained O_2_ delivery, thereby offsetting any reductions in blood flow secondary to possible apnea‐induced peripheral vasoconstriction (Tocco et al., 2013; Wein et al., 2007). This mechanism would ensure that there was sufficient O_2_ availability at the muscle to sustain the aerobic energy demand of the exercise.

Furthermore, the unchanged [La^−^] between free‐breathing and breath‐holding (Figure 2c), suggests a similar contribution from anaerobic glycolysis under both conditions and thus, no shift towards anaerobic glycolytic metabolism under the breath‐holding condition. Considering that identical power outputs, and thus exercise intensities, were used under both breathing conditions in the current study, the determining factor for elevated [La^−^] due to breath‐holding, compared to free‐breathing, would have been apnea‐induced hypoxia, and an increase in glycolytic phosphorylation to replace the reduced oxidative phosphorylation contribution (Woorons et al., 2014). Our data suggest that the short exercise duration of 20 s was not long enough to induce this hypoxia‐driven shift from aerobic to anaerobic glycolytic metabolism (Guimard et al., 2014, 2021). This concurs with studies of maximal speed 50 m freestyle swimming (~30 s) that also showed no differences in [La^−^] following breath‐holding compared to free‐breathing (Guimard et al., 2017, 2018). However, when 40 s apneas were performed during steady state cycling at 80 W, a workload that is not normally associated with lactate accumulation, there was a greater increase in [La^−^] compared to steady state cycling at 80 W while breathing normally, supporting the view that an increased anaerobic metabolic rate is associated with longer‐duration apneas (Andersson et al., 2004).

As expected, P_et_O_2_ was significantly lower under the breath‐holding condition (76 ± 14 mmHg), compared to free‐breathing (111 ± 11 mm Hg), immediately post‐exercise (Figure 2g). Although apnea likely resulted in a reduction in dissolved O_2_ stores, our data show that the short duration of 20 s was not sufficient to impair the required O_2_ demand for the exercise. Moreover, the apnea derived O_2_ deficit was repaid quickly when breathing resumed (Lindholm & Linnarsson, 2002) considering the increased V̇O_2_ compared to free‐breathing, post‐exercise (Figure 2d).

Another consequence of apnea is the inability to remove CO_2_ produced during the breath‐hold, resulting in CO_2_ accumulation (Linér & Linnarsson, 1994; Woorons et al., 2007), as was observed by the increased P_et_CO_2_ immediately post‐exercise (Figure 2h). In the present study, P_et_CO_2_ was 33 ± 6 mmHg post‐exercise under the free‐breathing condition, whereas under the breath‐holding condition it increased from 33 ± 4 mmHg pre‐exercise to 49 ± 7 mmHg post‐exercise, compared to 46 ± 1 mmHg following 75 s apnea at rest (Linér & Linnarsson, 1994) and 62 ± 1 mmHg following 40 s apnea while cycling at 80 W (Andersson et al., 2004). This increased P_et_CO_2_ is indicative of apnea‐induced hypercapnia and leads to discomfort, increased perceived exertion, and a strong desire to breathe (Guimard et al., 2018; Hubbard et al., 2024; Woorons et al., 2007). However, this CO_2_ accumulation was quickly expired when breathing resumed, as reflected by the increased V̇CO_2_ (Figure 2e) and V̇_E_ (Figure 2f), compared to the free‐breathing condition post‐exercise.

As no measure of the high‐energy phosphate system was taken in the present study, it is unknown whether there was an increased contribution from these high‐energy stores. However, considering breath‐holding did not result in greater O_2_ extraction, or anaerobic glycolysis, it appears that, aerobic energy production was not compromised and that there were sufficient intrinsic O_2_ stores to sustain aerobic energy production during short‐duration, high‐intensity exercise while breath‐holding.

### 4.2 Physiological response post‐exercise

Systolic BP was higher under the breath‐holding condition, compared to free‐breathing, immediately post‐exercise (Figure 2a). This may have been exercise‐induced, due to the increased cardiac output resulting from the increased HR during exercise, and/or apnea‐induced peripheral vasoconstriction. Furthermore, although apnea resulted in tachycardia during exercise, a bradycardia was observed post‐exercise under the breath‐holding condition, compared to free‐breathing (Figure 3a). This bradycardia may have been a function of the baroreceptor response due to the increased BP (Perini et al., 2008), or apnea‐induced hypoxia may have resulted in chemoreceptor‐driven bradycardia (Alboni et al., 2011; Wein et al., 2007). It appears that, although the exercise response prevailed during exercise, the apnea response may have had residual effects, post‐exercise, once the high‐intensity exercise stimulus was removed. In a comparison of 20 s bouts of cycling at 250 W under free‐breathing and breath‐holding conditions, it was found that HR and BP were higher under the breath‐holding condition, and it took longer for BP to recover under the breath‐holding condition, compared to free‐breathing (Hoffmann et al., 2005). The authors suggested a transient increase in vasoconstriction on cessation of exercise while breath‐holding in response to the apnea during exercise (Hoffmann et al., 2005). Our data suggest a similar response at a much higher power output.

The bradycardia persisted for 5 min post‐exercise in the current study (Figure 3a). As stated previously, most other studies it has been reported that HR returned to pre‐apnea levels quickly post‐apnea (Bergman et al., 1972; Bouten et al., 2020, 2021; Breskovic et al., 2011; Espersen et al., 2002), whereas one study observed a bradycardia compared to pre‐exercise baseline at 2.5 and 5 min of recovery following maximal duration apneas (126 ± 36 s) while kicking on a swimming ergometer at low‐intensity (18.1 ± 5.5 W) and HR had returned to baseline by 15 min of recovery (Brown et al., 2024). Our gas exchange data show that V̇O_2_ was lower during the second‐ and third‐min post‐exercise, and that V̇CO_2_ and V̇_E_ were lower from the second‐ through fifth‐min post‐exercise, under the BH condition (Figure 4). It is suggested that these responses are associated with residual effects of the apnea response, that is, bradycardia leading to reduced blood flow and thus reduced V̇O_2_ as well as V̇CO_2_ and the related V̇_E_. Collectively, these findings suggest residual cardiorespiratory effects of the apnea response to short‐duration, high‐intensity exercise while breath‐holding, and to our knowledge, this is the first time that this has been reported.

Finally, no differences were observed in these effects between males and females, which is in keeping with findings of similar responses between males and females during apnea at rest in which apnea‐induced HR reduction and O_2_ desaturation were not different between the sexes (Pernett et al., 2024). No sex differences in HR reduction have also been demonstrated during apneas in air, and with facial immersion in participants naïve to breath‐holding (Cherouveim et al., 2013) and breath‐hold trained divers (Peng et al., 2021), and comparable responses between sexes in stroke volume and cardiac output during apneas have also been reported (Peng et al., 2021; Tocco et al., 2012).

## 5 CONCLUSION

Although the apnea response has been shown to prevail over the exercise response during low‐intensity exercise in previous studies, in this study of high‐intensity exercise the exercise response prevailed over the apnea response. During high‐intensity exercise while breath‐holding, the exercise response may prevail over the apnea response due to the increased parasympathetic withdrawal and sympathetic drive of the high‐intensity exercise. Indeed, in our study, exercise‐induced tachycardia was observed rather than apnea‐induced bradycardia during 20 s high‐intensity exercise while breath‐holding. Had longer durations been used, a greater apnea‐induced bradycardia may have eventually overcome the exercise tachycardia. However, as there is an inverse relationship between exercise intensity and breath‐hold duration, breath‐holding during high‐intensity exercise may not be sustained for long enough for the apnea response to overcome the exercise response, as it does during low‐intensity exercise. Thus, when breath‐holding is necessitated during exercise, if breath‐hold duration is the priority (e.g., during free diving or breath‐holding competitions) efforts should be made to keep exercise intensity as low as possible. On the contrary, when high‐intensity efforts are required while breath‐holding (e.g., in sprint swimming races, artistic swimming competitions, or water rescue operations), these efforts may only be sustainable for short periods, and the higher the exercise intensity, the shorter the duration.

In the current study, a greater reliance on O_2_ extraction, or anaerobic glycolysis did not materialize, suggesting that intrinsic O_2_ stores were sufficient to sustain the aerobic energy contribution to high‐intensity exercise of this relatively short duration. Any potential reduction in O_2_ availability at the muscles due to apnea, or apnea‐induced peripheral vasoconstriction, may have been offset by increased O_2_ delivery due to the exercise tachycardia. Considering apnea under the conditions of the present study does not appear to alter energy production or impair performance during 20 s high‐intensity exercise, the biomechanical benefits of breath‐holding during sprint swimming should be considered viable. However, residual effects of an initial bout of whole‐body high‐intensity exercise while breath‐holding included reduced HR and gas exchange and ventilation post‐exercise. This may have an impact on subsequent bouts of exercise and as such should be borne in mind in situations where further exercise may be required following an initial bout of exercise while breath‐holding.

## AUTHOR CONTRIBUTIONS

J.W. and G.R.B. conceived and designed the research; J.W., J.L.R., N.A.G., and R.F. performed experiments; J.W. and G.R.B. analyzed data; J.W., G.R.B., and D.A.K. interpreted results of experiments; J.W., N.A.G., and R.F. prepared figures; J.W. drafted the manuscript; J.W., N.A.G., D.A.K., and G.R.B. edited and revised the manuscript and all authors approved the final version of the manuscript.

## FUNDING INFORMATION

Jeremy Walsh was supported by a National Sciences and Engineering Research Council of Canada (NSERC) Canada Graduate Scholarship—Doctoral grant (CGS D—579662‐2023).

## CONFLICT OF INTEREST STATEMENT

No conflicts of interest, financial or otherwise, are declared by the authors.

## Data Availability

The data that support the findings of this study are openly available in the Open Science Framework repository at https://doi.org/10.17605/OSF.IO/V9UCQ.
